# Characterisation of premature cell senescence in Alzheimer’s disease using single nuclear transcriptomics

**DOI:** 10.1007/s00401-024-02727-9

**Published:** 2024-05-02

**Authors:** Nurun N. Fancy, Amy M. Smith, Alessia Caramello, Stergios Tsartsalis, Karen Davey, Robert C. J. Muirhead, Aisling McGarry, Marion H. Jenkyns, Eleonore Schneegans, Vicky Chau, Michael Thomas, Sam Boulger, To Ka Dorcas Cheung, Emily Adair, Marianna Papageorgopoulou, Nanet Willumsen, Combiz Khozoie, Diego Gomez-Nicola, Johanna S. Jackson, Paul M. Matthews

**Affiliations:** 1 Department of Brain Sciences, Imperial College London, Hammersmith Hospital, Du Cane Road, London, W12 0NN UK; 2 Centre for Brain Research and Department of Pharmacology and Clinical Pharmacology, University of Auckland, Auckland, New Zealand; 3 Department of Psychiatry, University of Geneva, Geneva, Switzerland; 4 UK Dementia Research Institute Centre, Imperial College London, London, UK; 5 UK Dementia Research Institute Centre, King’s College London, London, UK; 6 School of Biological Sciences, University of Southampton, Southampton, UK; 7 Department of Basic and Clinical Neuroscience, Institute of Psychiatry, Psychology and Neuroscience, King’s College London, London, UK

**Keywords:** Aging, Senescence, Glia, Alzheimer’s disease, Microglia, Oligodendroglia, Astrocyte, Neuron, Single cell transcriptomics, Image mass cytometry, Cell stress, Senolytics

## Abstract

Aging is associated with cell senescence and is the major risk factor for AD. We characterized premature cell senescence in postmortem brains from non-diseased controls (NDC) and donors with Alzheimer’s disease (AD) using imaging mass cytometry (IMC) and single nuclear RNA (snRNA) sequencing (> 200,000 nuclei). We found increases in numbers of glia immunostaining for galactosidase beta (> fourfold) and p16^INK4A^ (up to twofold) with AD relative to NDC. Increased glial expression of genes related to senescence was associated with greater β-amyloid load. Prematurely senescent microglia downregulated phagocytic pathways suggesting reduced capacity for β-amyloid clearance. Gene set enrichment and pseudo-time trajectories described extensive DNA double-strand breaks (DSBs), mitochondrial dysfunction and ER stress associated with increased β-amyloid leading to premature senescence in microglia. We replicated these observations with independent AD snRNA-seq datasets. Our results describe a burden of senescent glia with AD that is sufficiently high to contribute to disease progression. These findings support the hypothesis that microglia are a primary target for senolytic treatments in AD.

**Supplementary Information:**

The online version contains supplementary material available at 10.1007/s00401-024-02727-9.

## Introduction

Alzheimer’s disease (AD) is the most prevalent form of late-life dementia. It is characterized neuropathologically by extracellular deposits of β-amyloid and intracellular neurofibrillary tangles accompanied by microgliosis, astrogliosis, markers of pan-cellular mitochondrial and lysosomal dysfunction and neurodegeneration [63, 74, 76]. Ageing is the greatest risk factor for AD [26, 53, 60] and is associated with cellular senescence. Accumulation of senescent cells in aging tissues can be accelerated by a wide range of cell stressors, including chronic inflammation [27].

Cell senescence characterized by an irreversible state of cell cycle arrest after proliferative cells reach the so-called “Hayflick limit” is known as replicative senescence [28]. However, premature senescence also is observed with chronic stressors in both proliferative and post-mitotic cells in association with chronic oxidative stress or mitochondrial dysfunction and the induction of DNA damage [5, 25, 35, 59]. Expression of a senescence-associated secretory phenotype (SASP) with senescence in either context can initiate inflammatory responses and propagate locally with release of paracrine mediators [14]. Increased lysosomal β-galactosidase (*GLB1*) and the DNA damage repair protein p16^INK4A^ (p16, encoded by *CDKN2A*) are commonly used immunohistological biomarkers of senescence [41].

Recent evidence has shown that pathological stressors can induce cell senescence in AD to levels above that seen with healthy aging. More than one mechanism may contribute to this. Aβ_1–42_ can induce senescence in astrocytes in vitro and greater numbers of p16-positive astrocytes have been reported in the frontal cortex of AD patients compared to that from non-diseased control brains [3]. Increased numbers of p16-positive astrocytes and microglia also have been reported with tau-mediated disease initiation and progression in a mouse model [8]. Associations of premature senescence with both oligodendrocyte progenitor cells (OPCs) [80] and microglia [32] in the APP/PS1 mouse and in postmortem AD samples have been described, along with evidence linking microglial senescence to increased replicative stress with aging and β-amyloid triggered generation of disease-associated microglia (DAM) [32, 49]. However, features of premature senescence associated particularly with DNA double-strand breaks (DSBs) and p16 expression also have been described in post-mitotic cells such as neurons[16, 18, 29].

Despite previous evidence of premature senescence of glia with AD, their aggregate burden, the cells most affected and the mechanistic triggers for senescence in them have not been well described. Here we have comprehensively characterized senescence in postmortem human brain tissue from entorhinal, middle temporal and somatosensory cortices of non-diseased control (NDC) and AD donor brains. We used convergent methods based on immunostaining and distinct transcriptomic analyses, as well as confirmatory analyses of publicly available transcriptomics datasets, to enhance confidence in our findings. We found evidence for increased senescence in AD brains compared to NDC in microglia and more partial senescence signatures in oligodendrocytes, and astrocytes, but little evidence for this in OPCs or neurons. We showed a direct association between β-amyloid accumulation and microglial senescence and provide evidence for its potential functional significance with evidence that senescence reduces transcriptomic signatures of phagocytosis for β-amyloid clearance. Finally, we distinguished mechanisms potentially responsible for initiating senescence pathways in the microglia as a foundation for the design of future senolytic therapies.

## Results

### Increased expression of markers of cellular senescence in glia in AD

**Fig. 1:**
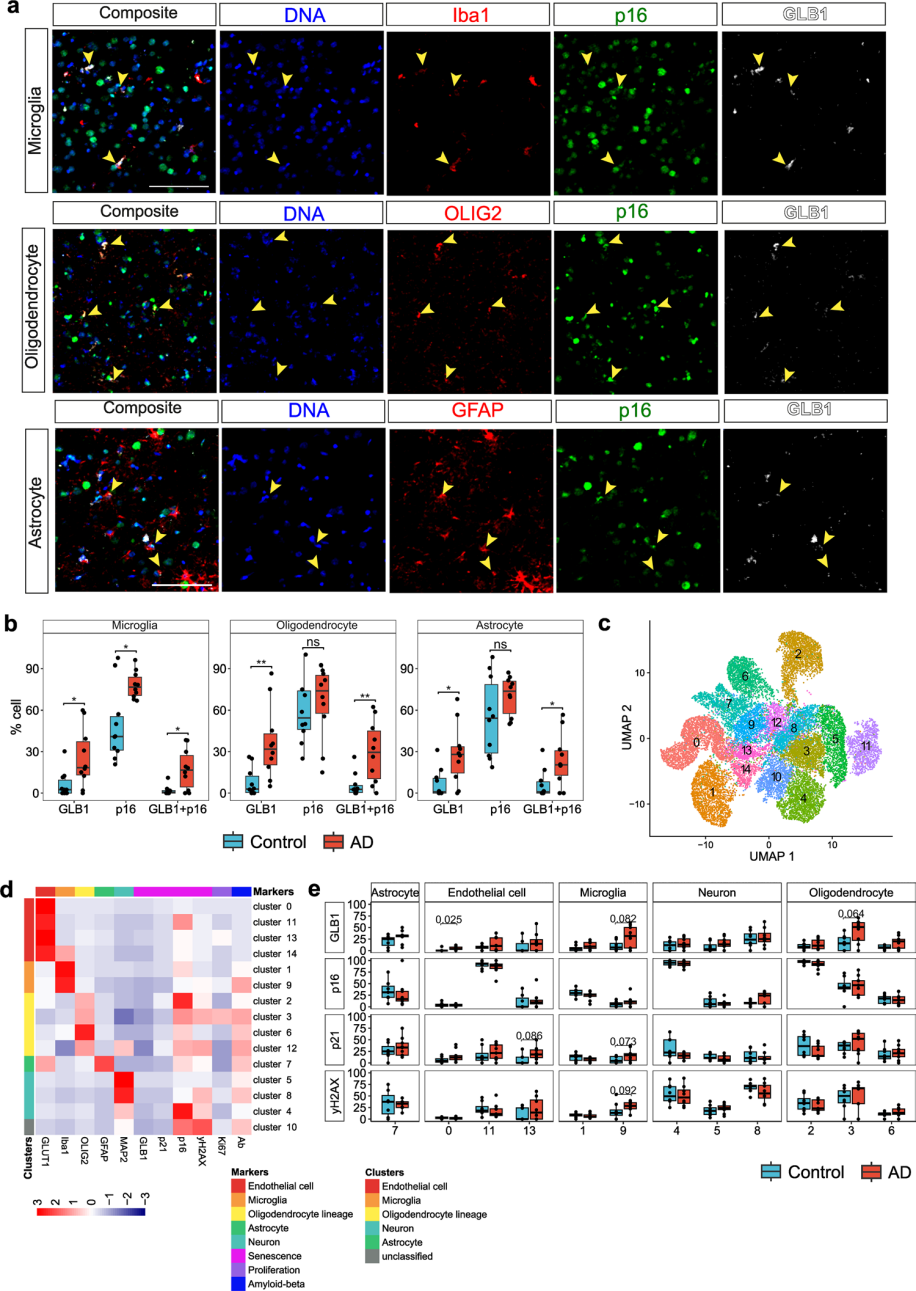
Numbers of senescent glial cells are increased in brains of AD compared to those from NDC donors. (**a**, **b**: IMC data from cohort-1) **a** Multiplexed imaging mass cytometry (IMC) (scale bar = 50 µm) revealed overlapping expression of senescence (GLB1 and p16) and cell type specific markers (Iba1; microglia, OLIG2; oligodendrocyte lineage, GFAP; astrocyte) in cohort-1. **b** Proportions of GLB1^+^, p16^+^ and GLB1^+^p16^+^ microglia (Iba1^+^), oligodendrocyte (OLIG2^+^) and astrocyte (GFAP^+^) were calculated in AD and NDC using cohort-1 IMC data (Wilcoxon rank-sum test, *p ≤ 0.05. **p ≤  0.01). (**c**–**e**: IMC data from cohort-2) **c** UMAP showing the cellular clusters generated by SIMPLI from cohort-2 IMC data. **d** Marker mean expression heatmap by clusters. **e** Proportion of nuclei of all clusters expressing senescence markers in AD and NDC (Wilcoxon rank-sum test, *p* ≤ 0.1 is reported)

We performed imaging mass cytometry (IMC) on postmortem cortical tissue from the middle temporal gyrus (MTG) of neuropathologically diagnosed AD and non-diseased control (NDC) donors of similar ages (Cohort-1: *n *= 10 from each group, NDC: Braak stage 0–II, AD: Braak stage V–VI, Supplementary data S1) to assess the co-localization of cell type specific protein markers (microglia, Iba1; oligodendrocyte lineage, OLIG2 [72]; astrocyte, GFAP; endothelial cell, GLUT1; neuron, MAP2) with GLB1, which encodes β-galactosidase, a marker for senescence-associated increases in lysosomal content [42], and with p16, a marker of DNA damage [51]. We found 4.1-, 4.6- and 4-fold more GLB1^+^ microglia (*p* value = 0.033), oligodendrocytes (*p* value = 0.0031) and astrocytes (*p* value = 0.02), respectively, in cortical tissue from donors with AD compared to NDC (Fig. 1a, b, Supplementary Fig. 1a-c, Supplementary data S1). We found a 1.6-fold increase in p16 positive microglia with AD compared to NDC (*p* value = 0.01) (Fig. 1b, Supplementary Fig. 1a–c) but no significant differences between numbers of p16 positive oligodendrocytes (1.1-fold, *p* value = 0.32) or astrocytes (1.3-fold, *p* value = 0.31) were found. No differences were found in the numbers of GLB1^+^ endothelial cells and GLB1^+^ neurons between AD and NDC (Supplementary Fig. 1d).

To understand the correlations between disease stages and activation of senescent pathways across cell types, we extended our findings by performing IMC with a larger panel of antibodies on a second cohort consisting of AD and non-diseased control (NDC) donors from a broader range of Braak stage (Cohort-2, NDC: Braak stage 0–II, AD: Braak stage III–VI, Supplementary data S2). We obtained data from entorhinal cortex (EC, which had the highest pTau/β-amyloid pathology load of the tissues studied from each brain), middle temporal gyrus (MTG, which had a moderate pTau/β-amyloid pathology load) and somatosensory cortex (SSC, which showed the lowest relative pTau/β-amyloid pathology load) from NDC (*n* = 8) and from donors with neuropathologically defined AD (*n* = 9). We included an antibody for p21^CIP1^ (p21, encoded by *CDKN1A*), that functions to maintain the viability of DNA damage‐induced senescent cells and one for γ-H2AX, a marker of DNA double-strand breaks (DSBs) [2, 79]. Because of the larger number of samples and antibodies in the panel, we used the automated image analysis method SIMPLI for processing this set of IMC images [7]. Using single-cell segmentation, we classified 19,947 cells which were grouped into 15 distinct cell clusters following unsupervised clustering (Fig. 1c). Based on the marker expression profiles, we assigned clusters to major cell types, excluding those expressing more than one cell type marker (Iba1^+^ microglia, cluster-1, 9; GFAP^+^ astrocytes, cluster-7; OLIG2^+^ oligodendrocyte lineage, cluster-2, 3, 6; MAP2^+^ neuron, cluster-4, 5, 8; GLUT1^+^ endothelial cells, cluster-0, 11, 13) (Fig. 1d).

We compared the proportion of cells expressing senescence markers (p16, p21, GLB1 and γ-H2AX) in each cluster and found that cluster-9 representing microglia (Iba1^+^) was both enriched for β-amyloid (Fig. 1d) and had an increased proportion of senescent nuclei expressing GLB1, p21 and γ-H2AX in AD compared to NDC in MTG following a similar trend as we observed in Cohort-1 (*p* value < 0.1, Fig. 1e, supplementary Fig. 2a). We found increased numbers of OLIG2^+^GLB1^+^ (cluster-3) in the MTG, similar to our findings in Cohort-1 (*p* value < 0.1, Fig. 1b, e). We also found increased numbers of cells expressing γ-H2AX and p21 in one neuronal cluster in the EC (cluster-5) (*p* value < 0.1, Supplementary Fig. 2b) providing evidence for responses to DSB in neurons independent of other senescence marker proteins. We did not find any cluster to be enriched specifically in AD compared to NDC (Supplementary Fig. 3a, b) suggesting the increased proportion of senescent microglia was not due to increased cell population. This confirmed a comprehensive profile of expression of senescence markers in microglia and suggested an association with β-amyloid in AD. Only a single senescence marker was expressed in oligodendroglia and markers of DSB (associated with oxidative DNA damage) were found only in a sub-population of neurons.

### Senescence-associated gene expression increases in microglia with AD

We performed single-nuclei RNA sequencing (snRNAseq) of cortical tissue from postmortem brains (Cohort-2, as used for the IMC shown in Fig. 1c–e, NDC: Braak stage 0–II, AD: Braak stage III–VI, Supplementary data S2) to extend our characterization of cell senescence phenotypes with AD.

**Fig. 2:**
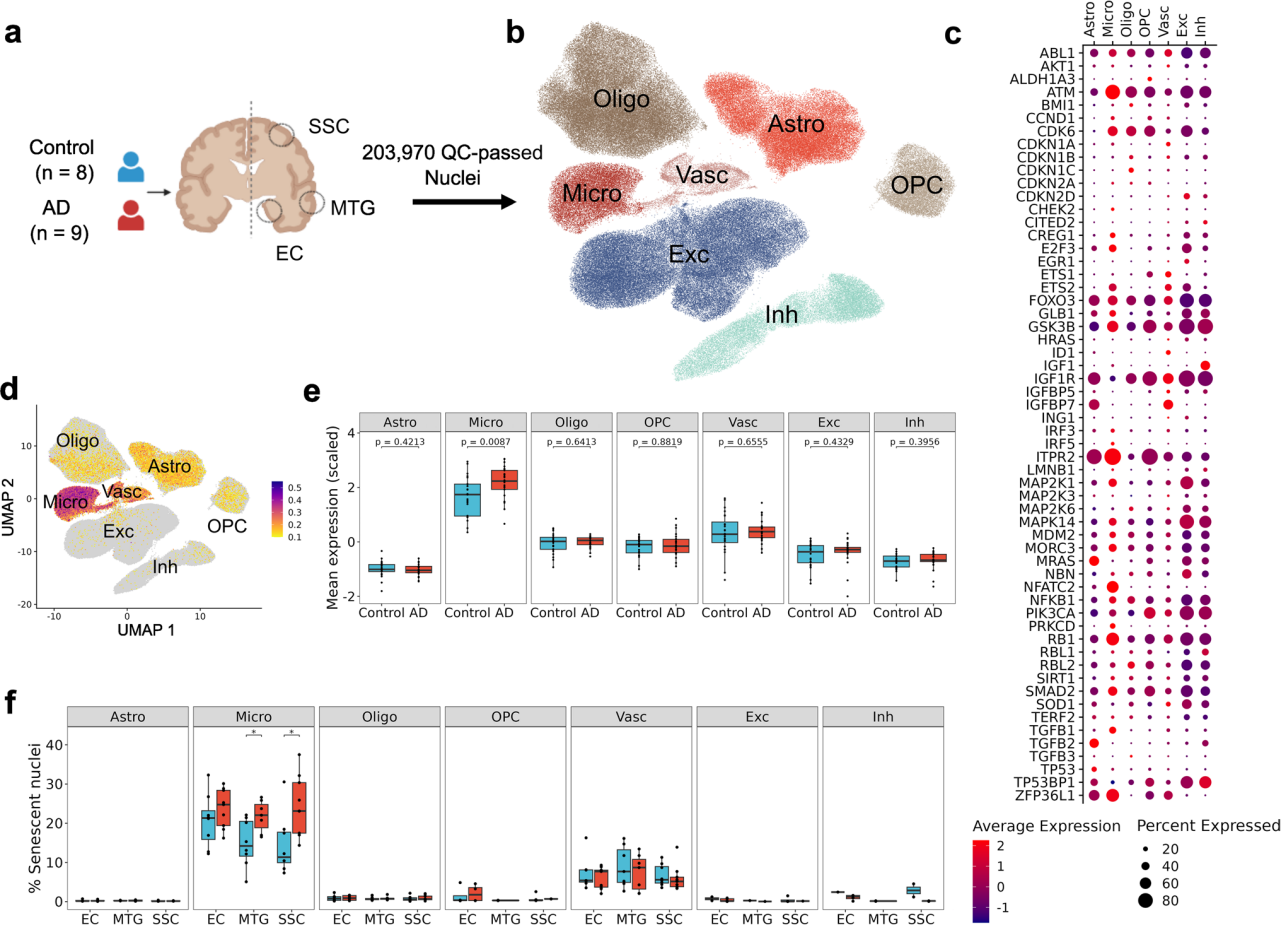
The microglial transcriptome is enriched for senescence gene expression in AD. **a** Study design showing the three regions from each of the 17 brains from which nuclei were isolated to generate snRNAseq data. **b** UMAP of seven distinct cell type populations characterized from snRNAseq data generated from 49 brain blocks. **c** Dot plot showing average scaled expression of genes and percentage of cells expressed from the ‘canonical senescence pathway (CSP)’, the ‘senescence initiating pathway (SIP)’ [16] and a custom senescence set (Supplementary data S5) for each cell type. **d** Normalised aggregated expression of genes as in **(c)** projected on UMAP. Color gradient scale showing aggregated gene set score in each nuclei. **e** Box plot showing scaled mean expression of the CSP genes in each cell type (Wilcoxon rank-sum test). **f** Percentage of senescent nuclei between AD and NDC across all cell types stratified by brain regions (Wilcoxon rank-sum test, *p ≤ 0.05) (Astro, astrocytes; Micro, microglia; Oligo, oligodendrocytes; OPC, Oligodendrocyte progenitor cells; Vasc, vascular cells; Exc, excitatory neurons, Inh; inhibitory neurons)

After rigorous quality control of the snRNAseq dataset (see Methods), 203,970 nuclei from seven major brain cell types were available for study (Fig. 2a-b, Supplementary Fig. 4a, Supplementary data S2). We assessed the relative enrichments of the “canonical senescence pathway (CSP)”, “senescence initiating pathway (SIP)” [16] and of senescence associated secretory phenotype (SASP) transcripts for each of the cell type specific clusters generated from the total set of AD and NDC brain nuclei. We found that the average expression of individual senescence gene was most in microglia (e.g., *ATM*, *RB1*, *NFATC2* and *GLB1*) (Fig. 2c). Astrocytes showed low levels of *TP53* (encoding p53) and *GLB1* transcript expression. Oligodendrocytes, OPCs and vascular cells showed the lowest expression of senescence pathway gene transcripts, with the exception of *CDKN1A* (encoding p21) which was highly expressed in vascular cells. Only very low levels of *GLB1* transcripts were expressed in neurons. These observations of individual genes were consistent with a relative enrichment for expression of the CSP in these cells: only microglia showed a significant (Wilcoxon test, *p* value = 0.0087) increase of CSP gene set expression in AD relative to NDC (Fig. 2d, e). We then calculated the proportions of nuclei expressing CSP (senescent nuclei) as described in [16] and found an increase in the proportion of senescent nuclei with AD only for microglia in the MTG an observation consistent with our imaging mass cytometry observations (Fig. 2f, Supplementary Fig. 4b). Additionally, increased proportion of senescent nuclei was also observed in SSC (Fig. 2f).

**Fig. 3:**
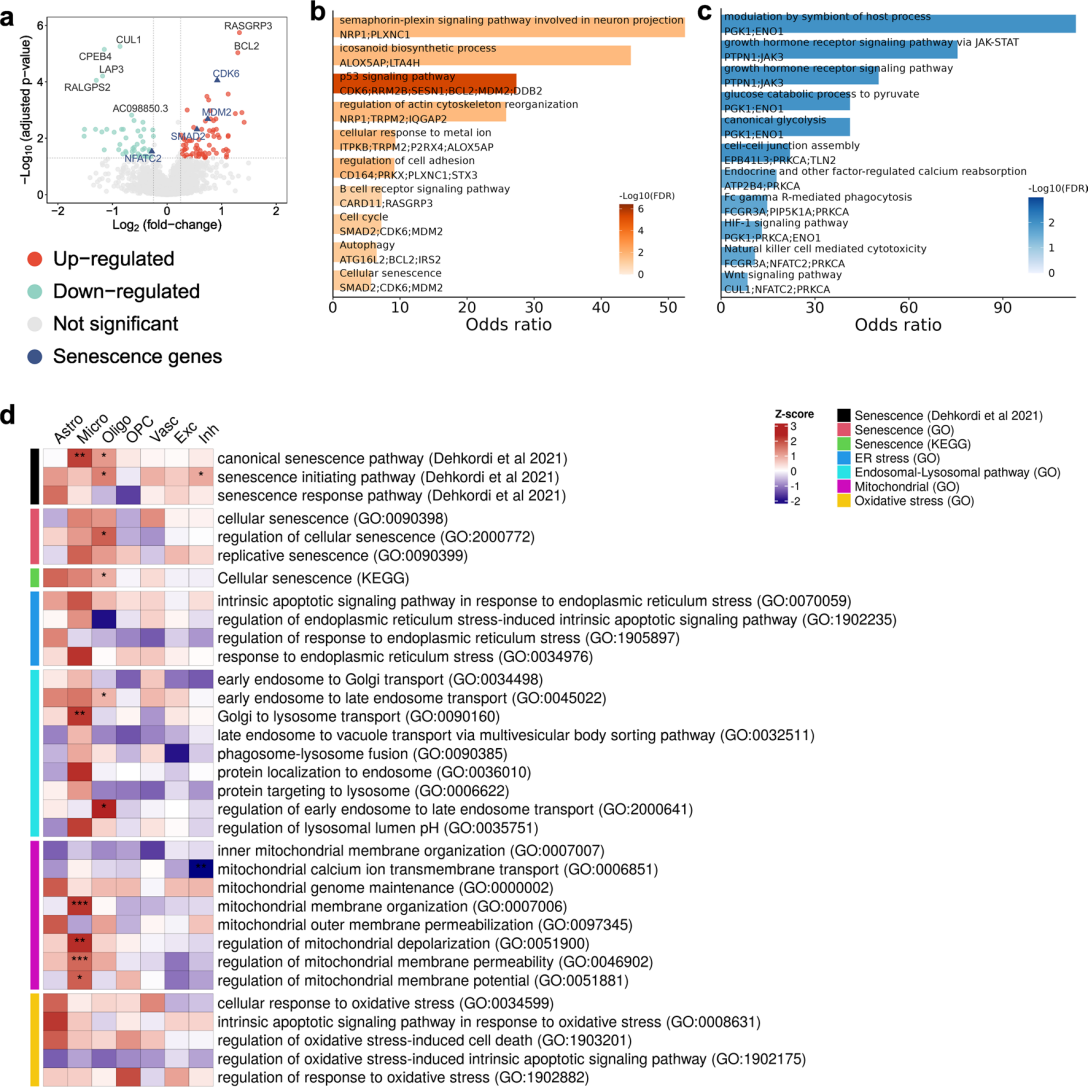
Senescence-associated genes are differentially expressed in microglia from AD and NDC donors. **a** Volcano plot showing differentially expressed genes between AD and NDC in microglia; senescence genes are annotated in blue. Barplots showing up- **b** and down-regulated **c** gene pathway enrichments in microglia with AD relative to NDC. **d** Heatmap showing senescence and other relevant gene set enrichments in AD relative to NDC (adjusted p, *p ≤ 0.05, **p ≤ 0.01, ***p ≤ 0.001)

We tested for increased, cell-specific expression of genes and pathways associated with chronic stress that could trigger the initiation of senescence in AD [59]. Differential expression analysis confirmed upregulation of DNA damage response genes (*DDB2, RRM2B*) and cellular senescence genes in microglia (*CDK6, MDM2, SMAD2*) with AD relative to NDC (Fig. 3a, b Supplementary data S3). Gene Ontology (GO) enrichment analysis defined upregulation of genes in microglia associated with the p53 signaling pathway, regulation of actin cytoskeleton reorganization and the positive regulation of phosphatidylinositol 3-kinase signaling in addition to those for the cellular senescence pathway (Fig. 3b, Supplementary data S4). Downregulated genes in microglia were enriched for phagocytosis-related pathways (Fig. 3c), consistent with previous reports describing reduced phagocytic capacity of senescent microglia with ageing [57]. Pathways involved in trophic (e.g., “axonal transport”, “synaptic plasticity”) and metabolic support (e.g., “cholesterol and lipid homeostasis”) for neurons were downregulated in astrocytes, oligodendrocytes and OPCs. We did not find enrichment of any senescence related pathways in neurons. Instead, transforming growth factor beta (TGFβ)-related and the neuronal apoptosis pathway genes were upregulated with AD (Supplementary Fig. 5a–f, Supplementary data S3, S4).

We then tested for relative senescence gene set enrichment across these cell types using previously defined senescence and related pathways (see Methods for a description of gene set selections) (Supplementary data S5). The “canonical senescence pathway (CSP)” was significantly upregulated in microglia and oligodendroglia and the “regulation of cellular senescence” pathway was significantly upregulated in oligodendroglia in AD compared to NDC. Pathways for “regulation of mitochondrial depolarization” and “regulation of mitochondrial membrane permeability” also were upregulated in microglia (Fig. 3d).

### Premature senescence in microglia with AD is associated with increased β-amyloid

**Fig. 4:**
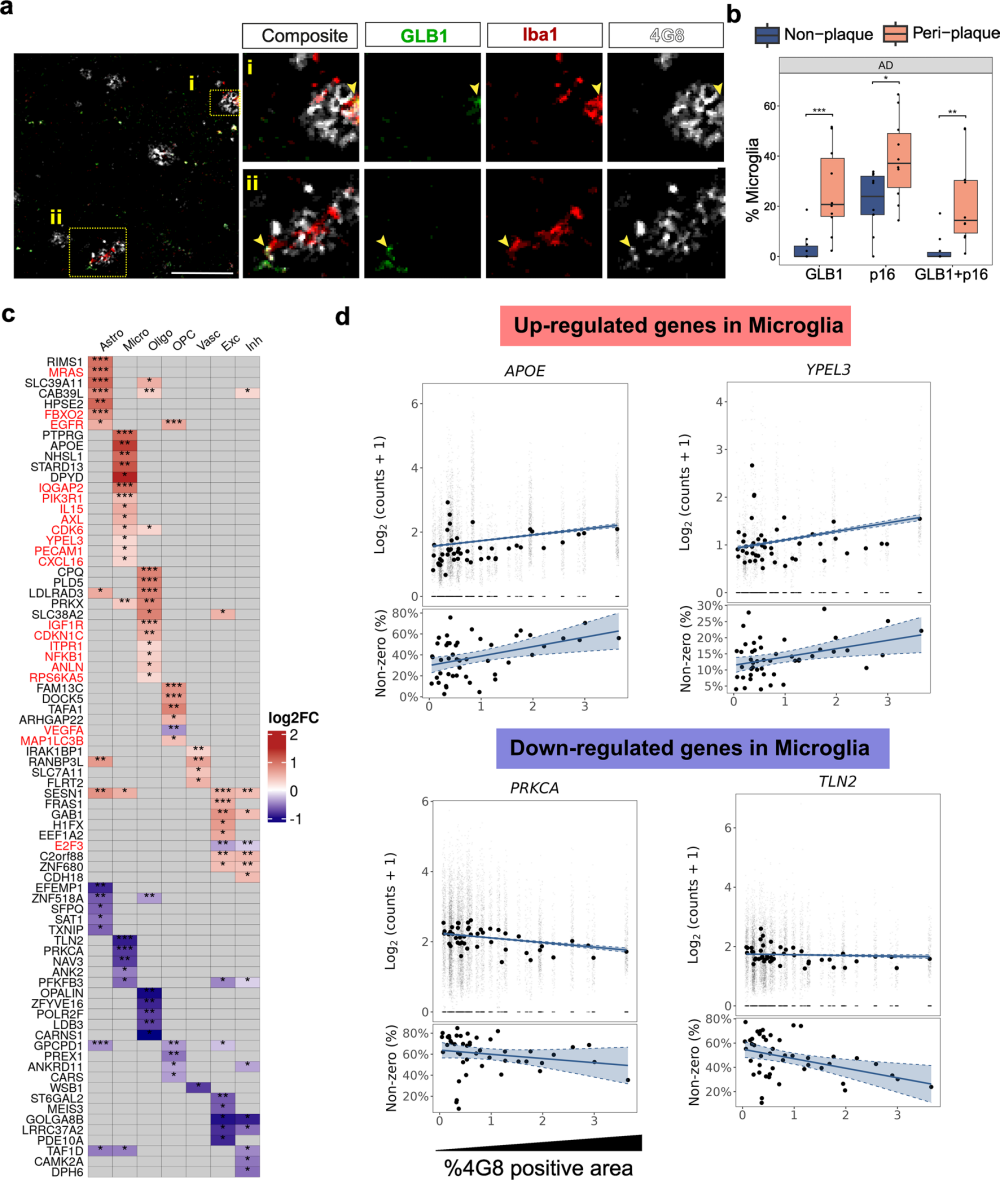
Senescence-associated genes are differentially expressed in microglia from AD and NDC donors. **a** IMC data (cohort-1) were used to quantify the number of cells positive for senescence markers GLB1, p16 or both by co-localizing them with the microglia marker Iba1 and 4G8^+^ β-amyloid plaque (yellow pixels). i and ii shows two plaque regions containing Iba1^+^positive cells. Iba1^+^ microglia within 10 μm of plaques were defined as peri-plaque microglia. **b** The proportion of peri-plaque microglia expressing senescence markers was significantly higher than for non-plaque microglia in AD (Wilcoxon rank-sum test, *p ≤ 0.05, **p ≤ 0.01, ***p ≤ 0.001). **c** Heatmap showing top 5 differentially expressed genes by logFC (adjusted *p* value 0.05) against β-amyloid loads measured by 4G8^+^ immunohistochemical staining densities in all cell types in snRNAseq data. **d** The gene expression graph illustrates its correlation with β-amyloid (4G8^+^) densities. The upper section displays the log2-normalized expression of each gene in individual nuclei, with median expression denoted by a black circle. The lower section indicates the percentage of non-zero nuclei in each sample

We hypothesized that the activation of senescence pathways in AD could be triggered by chronic oxidative stress from interactions with β-amyloid species [9]. Using our IMC images from cohort-1, we tested this hypothesis first by exploring whether microglia near β-amyloid plaques (peri-plaque, where highest exposures to β-amyloid may occur) more frequently express markers of increased senescence relative to microglia far from plaques (non-plaque). A co-localization analysis revealed more than 25% of microglia within 10 μm of β-amyloid plaques in AD showed expression of GLB1 while only 3% of microglia > 10 μm distant from plaques expressed this or other senescence markers (Fig. 4a, b, Supplementary data S1).

To validate an association between increased senescence and β-amyloid using the snRNAseq data, we performed a regression analysis for individual gene expression related to increasing loads (log_2_FC/% area) of immunostained (4G8^+^) β-amyloid in paired tissue sections from the contralateral (cryopreserved) hemispheres of each brain (Supplementary Fig. 13, Supplementary data S2) for each of the cell types. Transcripts for senescence associated genes including *YPEL3,* which encodes a protein downstream of p53 in the senescence pathway induced by DNA damage [36], *IQGAP2, IL15, AXL, PIK3R1, CDK6, PECAM1 and CXCL16* were significantly upregulated in microglia with increasing β-amyloid load (Fig. 4c, d) [61]. *GLB1* and *MRAS*, a senescence regulator, were upregulated in astrocytes. Oligodendrocytes also upregulated senescence associated genes (*CDK6, CDKN1C, ITPR1*), but we could not detect upregulation of genes associated with senescence in OPCs, neurons or in vascular cells with increased tissue β-amyloid (Fig. 4c, Supplementary data S6). We then tested for β-amyloid or Braak stage associated increases in senescence pathways in glia using gene set enrichment analyses. Microglia showed a significant upregulation of senescence, mitochondrial and endoplasmic reticulum (ER) stress-related gene sets for the “canonical senescence pathway (CSP)”, “senescence initiating pathway”, “regulation of cellular senescence” and “regulation of endoplasmic reticulum stress-induced intrinsic apoptotic signaling pathway” with greater β-amyloid and increasing Braak stage (Supplementary Fig. 6a–c, Supplementary data S5). Interestingly, “regulation of mitochondrial membrane depolarization”, which included *LRRK2* and *BCL2* genes, both of which can regulate apoptotic cell death pathways, was upregulated with increased β-amyloid [64]. Oligodendrocytes upregulated pathways for “senescence initiators”, “regulation of cellular senescence” and “intrinsic apoptotic signaling pathway in response to endoplasmic reticulum stress” and astrocytes upregulated the “senescence initiating pathway” (Supplementary Fig. 6a). Upregulation of CSP was found in oligodendrocytes when contrasted between AD and NDC (Fig. 3d) but not associated with β-amyloid suggesting the expression of full senescence phenotype in oligodendrocyte may not be pathology driven.

We did not find evidence for an increase in senescence gene set expression in OPC. We found little evidence for drivers of senescence of neurons, although we found a significant albeit to low level upregulation of “senescence initiating pathway” genes in inhibitory neurons (Supplementary Fig. 6a) with increased β-amyloid (Supplementary data S5). Together, these data thus describe comprehensive activation of a senescence transcriptional programme in microglia, expression only for senescence initiation pathways in astrocytes and oligodendroglia, and little evidence even for this amongst OPC and neurons.

We validated these observations using independent snRNAseq data for microglia and astrocytes enriched by FACS sorting. Differentially expressed transcripts in each cell type were identified by regression analysis against 4G8^+^ β-amyloid levels. We found increased numbers of senescence genes upregulated in both microglia (*YPEL3*, *IQGAP2, SQSTM1*) and astrocytes (*GLB1, SQSTM1, MRAS*) with greater levels of tissue β-amyloid densities [66] (Supplementary Fig. 7a, b, Supplementary data S7). Finally, we conducted a meta-analysis using published single-nucleus RNA sequencing (snRNAseq) datasets using the “canonical senescence pathway (CSP)” [16] gene set in microglia. Our analysis, which was based on 14 datasets, confirmed significant upregulation of this pathway in microglia for the vast majority of these (12/14, *p* value = 7e-04, Supplementary Fig. 7c, Supplementary data S8).

Senescence is a cell response to aging. We therefore formally tested for interactions between age and the β-amyloid pathology-associated senescence with AD in the datasets we used for the meta-analyses described above. First, we examined the NDC samples from our meta-analysis (who had an age range between 50 and 100 years old). Our findings revealed an increase in the expression of the CSP gene set with healthy aging in microglia; we found an apparent increase from 50 to 84 years of age in microglia (Supplementary Fig. 7d), to reach an apparent plateau. This finding is in agreement with an earlier report in which the authors calculated microglial cell-cycle length and concluded that human microglia reach the Hayflick limit for replicative senescence by the age of ~ 80 years [1]. However, when we compared microglia from donors with AD to those from NDC, the age association was no longer observed (linear-plateau modelling, Control; *p* value = 1.2e-05, AD; *p* value = 0.1). We interpret this as evidence that the dominant determinants of senescence in microglia with AD are disease-associated cell stressors (e.g., β-amyloid pathology) rather than chronological aging (Supplementary Fig. 7d).

### Identification of cell-specific triggers to initiation of senescence

We then explored biological pathways whose expression was enriched with increased β-amyloid load and these glial signatures of premature senescence or its initiation [10, 15]. We found lipid transport and homeostasis related inflammatory responses, cell migration, NF-kappa B signaling, p53 signalling pathway, regulation of cellular senescence, “negative regulation of oxidative stress-induced cell death” pathway upregulation and downregulation of G1/S transition of mitotic cell cycle in microglia, suggesting increased lipid metabolism, inflammatory response and cell cycle arrest were associated with premature senescence in response to greater β-amyloid load. Both oligodendrocytes and astrocytes upregulated genes enriched for lipid metabolism and NF-kappaB signaling pathways. However, the pathways expressed varied between cells, e.g., upregulated genes in oligodendrocytes were associated with interleukin-1-mediated signaling pathway, while those in astrocytes were associated with more general reactive and migratory astrocytic responses (e.g., *ACTN4, EGFR, SASH1*) [43, 78]. (Supplementary Fig. 8a, b, Supplementary data S9). In summary, microglia exhibited upregulation of pathways related to β-amyloid response, cell proliferation and migration, and senescence while oligodendrocytes upregulated lipid metabolism and NF-kappaB signaling pathways related to cellular inflammation and astrocytes showed evidence only for general activation and migration.

### Sub-populations of microglia and oligodendroglia are differentially susceptible to premature senescence with AD

Glia dynamically adopts different cell states that can be transcriptomically and functionally distinguished. We hypothesized that sub-populations of glial are differentially susceptible to premature senescence with AD. To test this, we subclustered microglia, oligodendrocytes and astrocytes into transcriptomic sub-populations distinguished by previously described marker genes [23, 33, 58].

**Fig. 5:**
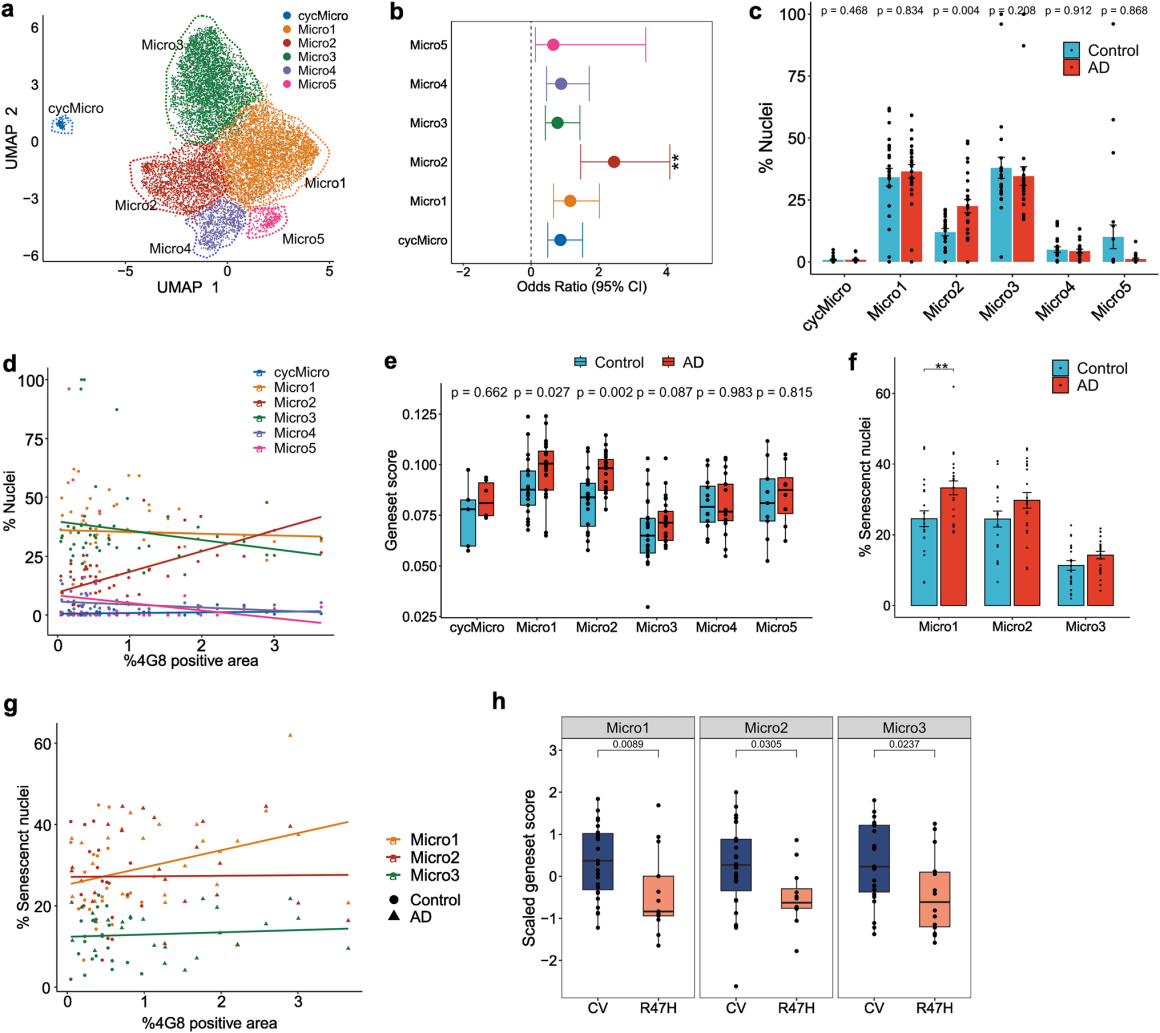
Sub-populations of microglia differentially express senescence gene signatures. **a** UMAP dimensionality reduction plot showing microglial sub-populations **b** Odds-ratio estimates of microglial sub-populations associated with AD (circle; OR estimate obtained from MASC [22], bars; 95% CI). **c** Differences in relative numbers of each of the microglial sub-populations isolated from AD and NDC cortical brain tissue. **d** Percentages of microglial sub-populations as a function of β-amyloid (4G8^+^) load (Micro2 Pearson’s R = 0.63, *p* value = 1.4e^−06^). **e** Boxplot showing normalised mean expression of the CSP gene set in the different microglial sub-populations (Wilcoxon rank-sum test). **f** Bar plots comparing the proportions of senescent nuclei between AD and Control for Micro1-3 (Wilcoxon test, **p value<0.01). **g** Correlations of the proportions of Micro1-3 senescent nuclei with β-amyloid (4G8^+^) immunostained areas in sampled region of each brain (Micro1 Pearson’s *R* = 0.34, *p* value = 0.021). **h** Boxplots showing the scaled mean expression of CSP gene set across microglial sub-population grouped by TREM2 genotype (CV, common allele, or the R47H AD risk variant)

We identified six microglial sub-populations. Micro1, Micro2, Micro4, Micro5 sub-populations corresponded to previously identified HM_0, GPNMB_EYA2/LPL_CD83, CRM_CCL3 and HM_4 microglial phenotypes, respectively (Fig. 5a, Supplementary Fig. 9a–c) [23]. Micro3 showed similarities to HM_3, HM_4 phenotype but also expressed *APOE*, apoptosis associated genes *XAF1, YWHAB, YWHAG, YWHAE* encoding 14–3-3 protein subunits, cell-cycle and cell division control genes *CDK12, MIS18BP1, TAF1D, BOD1L1* and *CCAR1* [6, 30, 67]. This microglial nuclei sub-population expressed fewer total genes and an increased percentage of mitochondrial genes relative to other sub-populations (Supplementary Fig. 9a, d, e) [54]. We also identified microglia population (cycMicro) that appeared to be actively proliferating as defined by the expression of multiple centromere proteins (*CENPF, CENPK, CENPU*), mitotic checkpoint kinase (*BUB1B*) and regulator (*CLSPN*), similar to the proportion estimated previously to be in S phase by Ki67 immunohistology [1] (Fig. 5a, Supplementary Fig. 9a; Supplementary data S10). The relative numbers of Micro2 nuclei were increased both with AD and with increased amyloid levels (Fig. 5b; logistic mixed-effect model, **p value ≤ 0.01, absolute log2(odds ratio-OR) > 2, Fig. 5c, Wilcoxon rank-sum, *p* value = 0.004, Fig. 5d; Pearson’s correlation *R* = 0.63, *p* value = 1.4e-06).

The senescence gene set was more highly expressed in Micro1 and Micro2 nuclei from AD brains relative to controls (Fig. 5e). This was not found for other sub-populations. We then calculated the fractions of individual nuclei in each sub-population that were enriched for CSP gene set expression [16]. The fraction of Micro1 nuclei enriched for CSP gene sets in AD was greater than in control tissue (Fig. 5f, Wilcoxon rank-sum, *p* value < 0.01) and this fraction increased in Micro1 nuclei in proportion to the tissue β-amyloid loads (Fig. 5g, Pearson’s correlation *R* = 0.34, *p* value = 0.021). Higher proportions of nuclei from the MTG expressed senescent gene sets than from the EC or SSC (Supplementary Fig. 9 f, g). Together, these analyses thus provide evidence for increased senescence with AD and increasing β-amyloid specifically amongst that HM (Micro1) and GPNMB_EYA2/LPL_CD83 (Micro2) sub-populations.

Finally, to test whether senescence in AD depends specifically on microglial activation with β-amyloid, we generated snRNA transcriptomes of microglia obtained from neuro-pathologically defined AD donors carrying the *TREM2 R47H* variant allele, which is associated with reduced responsiveness of microglia to β-amyloid [68, 83]. We found a reduced senescence gene signature in microglia from donors carrying the *TREM2 R47H* variant allele relative to the *TREM2* common allele, consistent with our hypothesis of β-amyloid driven senescence activation (Fig. 5h). We did not detect differences in the senescence gene set expression in nuclei from these donors when stratified by either *APOE* or *CD33* genotype (Supplementary Fig. 9h, i). As TREM2 acts as a β-amyloid receptor, this suggests that drivers of senescence in microglia are downstream of β-amyloid induced microglial activation.

**Fig. 6:**
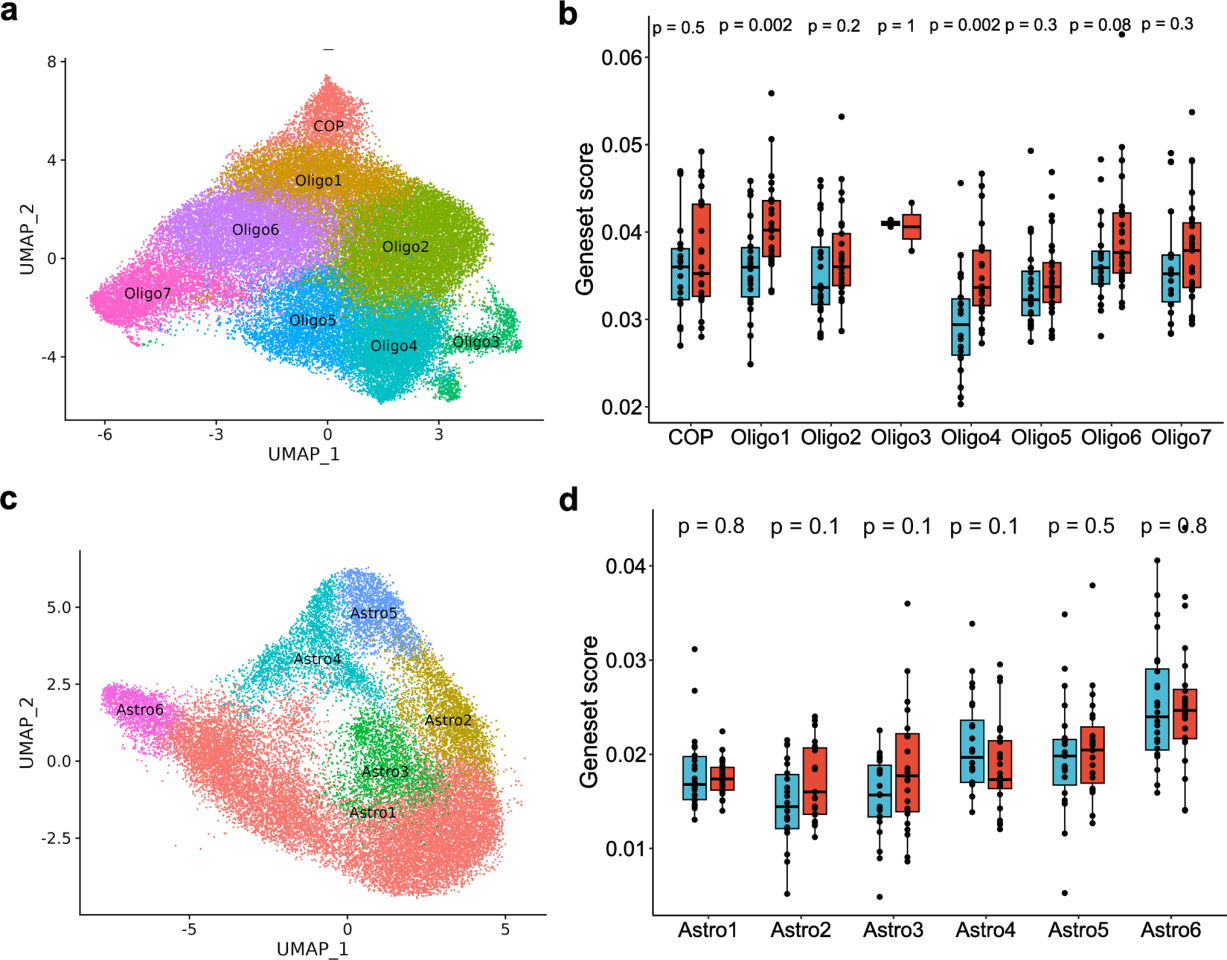
Sub-clustering of oligodendrocytes and astrocytes. Sub-clustering of oligodendrocytes **a**, **b** and astrocytes **c**, **d** show diverse functional sub-populations. UMAP dimensionality reduction plots describing these sub-populations for oligodendroglia **(a)** and astrocytes **(c)**. Normalised mean expression of CSP between AD and Control across sub-populations of oligodendrocytes **(b)** and astrocytes **(d)**

We used the same approaches to explore senescence in oligodendrocyte and astrocyte sub-populations (Fig. 6, Supplementary Fig. 10, Supplementary data S10). There were seven distinguishable sub-populations of oligodendroglia in addition to the committed oligodendroglia precursors (COP). We compared the transcriptomics profile of our oligodendrocyte sub-populations with those from the previous studies [33, 48]. Oligo1 and Oligo2 showed similarities with COPs and OPCs suggesting these subpopulations are not fully matured oligodendrocytes (Fig. 6a, Supplementary Fig. 10a, b). While Oligo3 represents a mature myelin forming oligodendrocytes, Oligo4 and Oligo7 resemblance to a mature but non-myelin forming oligodendrocytes. Finally, Oligo5 shows similarities to the end state of oligodendrocyte maturation as identified in [33]. Interestingly, Oligo1 and Oligo4 which showed signatures of imOLGs have increased senescence gene set expression with AD (Fig. 6b). As we found for microglia, specific sub-populations of immature oligodendroglia had evidence for increased senescence gene set expression, although we could not find a specific association with β-amyloid load.

Finally, we also explored senescence pathway expression in the six astroglial sub-populations that we described previously [66], but did not find that any of these sub-populations showed greater expression of the senescence gene set with AD (Fig. 6c, d, Supplementary Fig. 10c).

### Mechanisms of microglial activation by β-amyloid promote premature senescence

Senescence and apoptosis both can be triggered by aging and chronic cell stress [13, 24]. To more specifically explore whether senescence with AD includes a contribution from pathology independent of age, we directly correlated the relative proportions of microglia sub-population with age in AD and NDC. We found that numbers of Micro3 nuclei, which most highly expressed apoptotic pathway genes, decreased with increasing age in the NDC brain tissues, as expected for a sub-population prone to apoptosis. However, this was not observed in AD, consistent with induction of senescence pathways and reduced apoptosis (Supplementary Fig. 11a). To further validate this with an independent dataset, we integrated microglia nuclei from all the datasets used in the meta-analysis and used “transfer labelling” according to our subcluster annotation and found again that the proportion of Micro3 nuclei decreases with increasing age in NDC but not in AD samples (Supplementary Fig. 11b, Supplementary data S11). This suggests, AD-associated pathology can trigger senescence as a reversal of apoptosis phenotype.

To define specific mechanisms conferring vulnerability to senescence in microglia with AD, we explored differential gene expression in the Micro1, Micro2 and Micro3 sub-populations with AD relative to that in the NDC and with regression against β-amyloid load across all tissues studied, regardless of disease association. Our first level of analysis better defined mechanisms responsible for the induction of senescence. All three microglial sub-populations showed upregulation of *APOE, ATG16L2* (an autophagy antagonist gene), *BCL2* (encoding a negative regulator of apoptosis), the CDK7 regulator gene *CCNH* and *GAS7* (the growth arrest-specific protein 7 gene). Genes for negative regulators of senescence such as *GRB2* and *NIBAN1* were downregulated with increasing β-amyloid [17, 21, 34, 55]. The Micro3 subpopulation increased *TAOK1* expression, the protein product of which is involved in mitotic G2 DNA damage checkpoint signalling and the expression of genes for the positive regulators of senescence OXR1 (Oxidation Resistance 1) and PELI1 [46, 81] (Supplementary data S12).

**Fig. 7:**
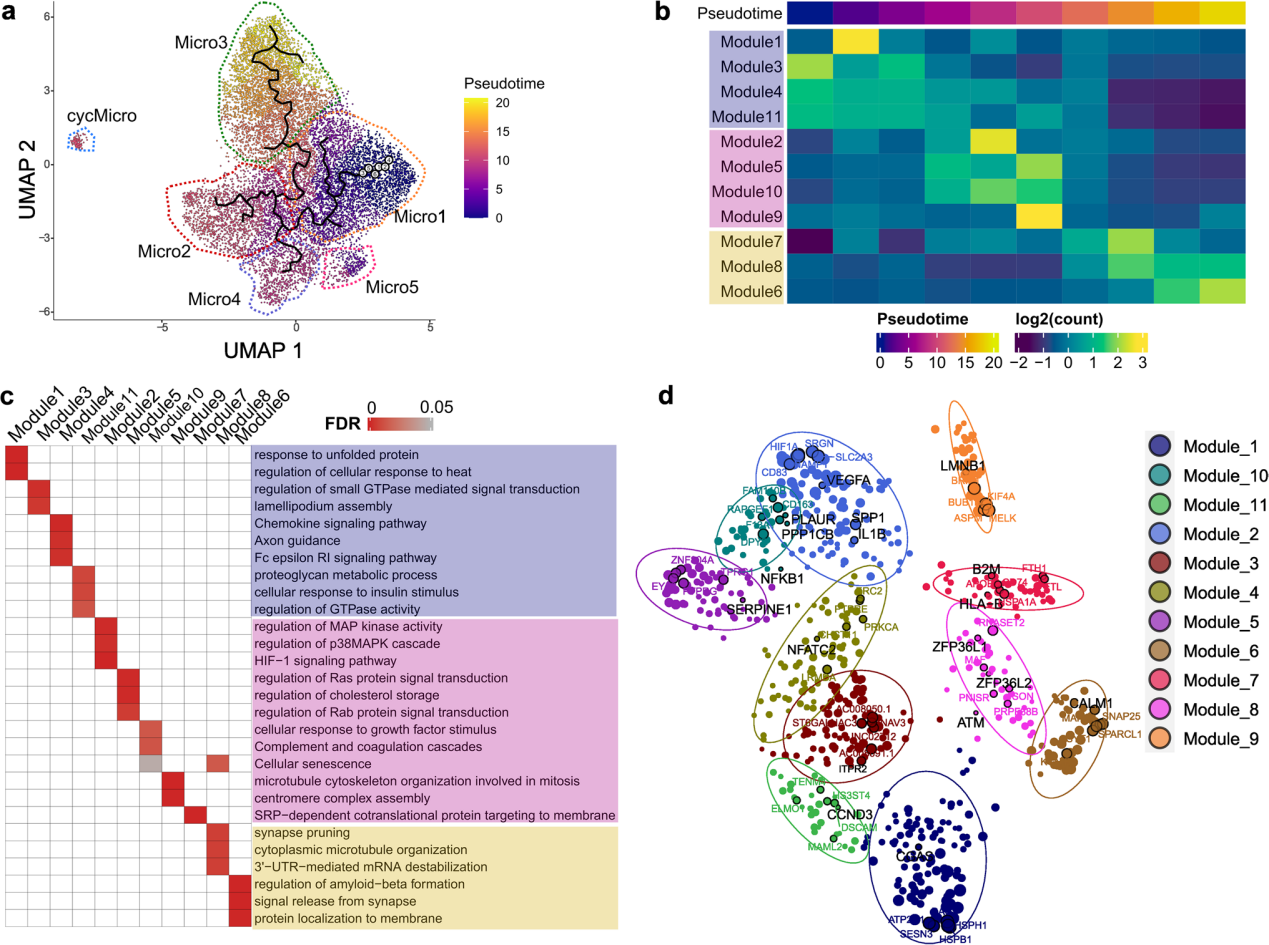
Premature senescence of microglia with greater pseudo-time in AD is associated with gene signatures for inflammatory activation. **a** UMAP plot colored by microglial pseudo-time trajectory calculated by Monocle3 [71]. **b** Heatmap showing the relative enrichment of gene co-expression modules differentially expressed with increasing pseudo-time. Module names are colored according to pseudotime progression: Purple, early-; lilac, mid-; yellow, late-pseudotime **c** Significantly enriched pathway of the co-expression module genes. Pathway names are colored according to plot **(b)**. **d** Network plot showing hub genes for each module (distinguished by colours) with senescence pathway associated genes highlighted (black circle)

To further characterize associated gene expression pathways from which triggers for senescence induction could be inferred, we generated disease-related pseudo-time trajectories from the total set of control and AD nuclei. We set Micro1 as the start of the pseudo-time as it most highly expressed homeostatic markers such as *P2RY12* and *CX3CR1*. Homeostatic (Micro1) to GPNMB_EYA2/LPL_CD83 (Micro2) and CRM_CCL3 (Micro4) responses were found to be two independent activation routes mirroring the observations in a recent pre-print [47]. Micro3 branched into a third route of the trajectory (Fig. 7a). Our pseudo-time analysis identified genes that were differentially expressed along the pseudo-time trajectories. These genes were grouped into gene co-expression modules which characterized the transcriptomic identity of the microglial sub-populations along the trajectory. (Supplementary Fig. 12a, Supplementary data S13). Functional pathways including “lamellipodium assembly”, “Fc-gamma receptor signalling pathway involved in phagocytosis” and “chemokine signalling pathway” (were enriched in the Module-3 and Module-4 genes that found predominantly in early-pseudotime suggesting early microglial activation in response to β-amyloid accumulation (Fig. 7b, c, Supplementary data S13). Module-5, which was highly expressed in mid-pseudotime, included GPNMB_EYA2 associated genes *GPNMB, PLA2G7, MYO1E, SLC1A3, CPM*, and *NPL* and pathways involving “regulation of macrophage derived foam cell differentiation”, “cellular response to low-density lipoprotein particle stimulus” and “positive regulation of inflammatory response” suggesting a β-amyloid associated pro-inflammatory and potentially harmful phenotype (Fig. 7c, Supplementary Fig. 12a). We found that SERPINE1, a known senescence marker and SASP factor, was co-expressed with these genes in Module-5 [73]. Regression analyses showed that module-5 gene set correlated strongly with increasing β-amyloid (Supplementary Fig. 12b, Supplementary data S14). This suggests that microglial pro-inflammatory activation and senescence phenotype are downstream of β-amyloid response. Module-10 also was highly expressed in Micro2, which was enriched for “NF-kappa B signalling pathway”, “mTOR signalling pathway” and “cellular senescence” pathways (Fig. 7c, Supplementary Fig. 12a, Supplementary data S13). Module-8, which also showed enrichment for cellular senescence pathways, was most highly expressed in Micro3 and in late-pseudotime (Supplementary Fig. 12a). *ZFP36L2, ZFP36L1, ATM, CX3CR1* and *C3* were amongst the genes highly expressed in module-8 (Fig. 7d). ZFP36L2, *ZFP36L1* are associated with SASP and *ATM* is known to be the key driver of DNA-damage induced senescence [82]. The Micro3 sub-population also highly expressed module-7 genes such as *CD74, APOE, FTL* and *FTH*, reflecting functional activation for chemokine-mediate migration and an immunosuppressive or pathological immune “exhaustion” (dystrophic) phenotype with greater β-amyloid load (Supplementary data S13). The co-expression of microglial functional activation and DNA damage gene expression and senescence transcripts in Micro3 suggest that β-amyloid induced activation can increase pro-survival phenotype in an otherwise apoptotic cell. Together, these trajectory analyses support the hypothesis that the senescence phenotype in microglia is expressed as part of a microglial inflammatory activation response to β-amyloid.

## Discussion

Although recent evidence has identified premature cell senescence in AD brains [59], we are not aware of a comprehensive characterization of which cell types are most affected, their relative numbers or the mechanisms responsible [5, 25, 35]. Here, we have used a combination of snRNA sequencing and imaging mass cytometry to characterize cell senescence in AD, describing cell types affected, the association between senescence and AD pathology and providing transcriptomic evidence for mechanisms of senescence. Our results demonstrate that there is a substantial burden of senescent cells in brain cortical grey matter with AD and that cell senescence signatures are most prevalent in microglia and associated with inflammatory activation and downregulation of genes related to β-amyloid phagocytosis. We also provide evidence that microglial senescence in AD is related to β-amyloid by showing greater proportions of senescent microglia near β-amyloid plaques and with greater β-amyloid load, particularly in the sub-population of microglia most enriched for homeostatic markers (Micro1) and DAM-associated genes (Micro2). Additional supportive evidence of β-amyloid induced senescence have come from demonstration of the TREM2 genotype dependence of the senescence signature (see also recent preclinical evidence presented in pre-print [56]). An intriguing aspect of our results is the observed lack of senescence in carriers of the TREM2-R47H variant. Contrary to expectations, this absence of senescence does not confer protective effects, as senescence may not be the predominant phenotype in microglia carrying R47H mutations. Instead, our data suggest that the overall pathology burden associated with R47H, particularly in the context of reduced microglial activation, may overshadow any potential neurotoxic effects attributed to senescent microglia. Our data additionally suggest mechanisms that DNA damage, mitochondrial dysfunction and ER stress associated with inflammatory activation together confer vulnerability to premature senescence in microglia with AD. These associations are supported by pseudo-time analysis that demonstrates the relationships of expression of these pathways with senescence along the inferred progression of microglia towards the alternative end states of apoptosis or senescence, expression of which may be related to the severity and chronicity of the stressors responsible [59].

Previous studies have highlighted senescence in multiple brain cell types in AD [3, 16, 32, 52, 80]. Most have focused on single or small numbers of markers of senescence, which likely accounts for some differences in conclusions that have been drawn. We employed multiplexed immunohistology and transcriptomics analyses of postmortem human brains to assess senescence more comprehensively. This highlighted the complexity of profiling senescence-related cell phenotypes. For example, we found increased levels of *GLB1* protein expression [19, 75] in microglia, oligodendrocytes and astrocytes. However, as GLB1 expression alone cannot distinguish between senescent cells and cells with intrinsically high lysosomal content and is less associated to senescence in post-mitotic cells such as neurons, we also employed p16 and p21 as markers of an arrested cell cycle and γH2AX to detect DNA double-strand breaks (DSBs) [41, 52]. We found microglia to be the only cell type with significant increase in all markers in AD. Microglia, oligodendrocytes and astrocytes transcriptomes all showed enrichment for genes upregulated in an early senescence (“senescence initiators”) pathway, but only microglia showed full expression of the full canonical senescence pathway (CSP) [16]. By this criterion, only microglia showed strong evidence for premature senescence with AD. Our data suggest mitochondrial dysfunction and ER stress are common mechanisms driving the premature microglial senescence in AD, but does not directly implicate impaired autophagy, as suggested by a recent preclinical study [69]. However, this deserves further investigation as it may reflect limitations of the range of cell processes, we were able to interrogate. Also mitochondrial dysfunction can lead to ER stress through impaired autophagy [4].

While we identified Olig2^+^ cells expressing some senescence markers, joint use of transcriptomic data was able to more probably ascribe this senescence signature predominantly to oligodendrocytes rather than OPC, which had been implicated in an earlier preclinical study [80]. Oligodendroglial cells also increased expression of ER and related cell stress pathway transcriptomes with increasing β-amyloid load. We found only a smaller, more partial senescence response associated with DSB identified by γH2AX and DNA damage associated pathway expression in inhibitory neurons specifically. Dehkordi et al. previously concluded that excitatory neurons uniquely expressed a senescence signature based on relative enrichment of nuclear genes expressed with an eigengene reflecting a canonical senescence response [16]. Our different conclusion likely reflects the lower specificity of the eigengene approach relative to the simultaneous application of the multiple approaches here, but also could be influenced by differences between the brains sampled in two studies, differences in pathological features correlated (early β-amyloid pathology here vs later phospho-Tau pathology in the earlier paper) and relative weights of the marker gene expressed (i.e., use of CDKN2D in excitatory neurons [16]). In line with this, we found an apparent difference in senescence cell burden between allocortex (EC) and neocortex (MTG, SSC).

The strengths of our study were that we applied both immunohistological and transcriptomic analyses to identify cells expressing senescence markers with AD and that we included analyses of regions showing different levels of AD pathology in each brain. However, like all previous studies of AD, our analyses described markers associated with senescence rather than providing direct evidence of permanent growth arrest for the potentially replicating glia. Processes leading to senescence also evolve dynamically, as our results showing varying degrees of partial senescence phenotypes with pseudo-time imply. Describing a dynamic process using a cross-sectional sampling strategy can lead to strong biases in inferences particularly regarding the proportion of end stage events. This could introduce bias particularly towards underestimates of the senescent cell burden. Cell senescence also is related to aging, as we showed for the non-diseased control brains. Our analysis of the contribution of aging to senescence in AD (either directly or through interactions) was limited by the low range of ages in the donor population. As aging is the strongest risk factor for AD, this deserves further study. Finally, the power to detect differentially expressed genes with AD or β-amyloid depends strongly on numbers of independent samples [62]. While 49 different brain blocks were characterized for our study, the three regions studied from each brain are not fully independent. This limits confidence particularly for single gene identifications. We mitigated this by replication of key observations with independent datasets, by highlighting results in agreement with prior studies that this work was extending and by analyses of gene sets, for which power should be substantially higher. Nonetheless, more work needs to be done to more confidently define specific genes whose expression appears to drive the pathology.

Preclinical studies using different models have suggested that senolytic treatments can reduce the abundance of senescent cells, disease-relevant pathology and cognitive deficits [8, 56, 80]. The extent to which cell senescence could contribute either to susceptibility to or the progression of human AD is important to answer fully in assessing the therapeutic potential of senolytics for this indication. Our study describes extensive cell stress associated with premature senescence in microglia in established AD. It additionally highlights that, by reducing phagocytic gene signatures in microglia in association with the induction of premature senescence, β-amyloid could reduce its own clearance and potentiate disease progression. Based on our observations, we hypothesise that microglia should be a primary focus for senolytic treatments in AD. They might be used synergistically with other strategies to enhance clearance of β-amyloid pathology [11]. This therapeutic opportunity provides a rationale for further characterization of mechanisms responsible for premature senescence in microglia.

## Methods

### Brain tissue

This study was carried out in accordance with the Regional Ethics Committee and Imperial College Use of Human Tissue guidelines. Cases were selected based first on neuropathological diagnosis from UK brain banks [London Neurodegeneration (King’s College London) and Parkinson’s UK (Imperial College London) Brain Bank]. We then excluded cases with clinical or pathological evidence for small vessel disease, stroke, cerebral amyloid angiopathy, diabetes, Lewy body pathology (TDP-43), or other neurological diseases. Where the information was available, cases were selected with a postmortem delay of less than 60 h (Supplementary data S1, S2). Cohort-1 included MTG cortical tissue from 10 donors (Braak stage 0–II) without evidence of clinically significant brain disease (non-diseased controls, NDC) and 10 neuropathologically defined AD (Braak stage V–VI) donors (Supplementary data S1). Cohort-2 included EC, MTG and SSC cortices from a final set of 17 cases including 8 NDC donors (Braak stage 0–II) and 9 neuropathologically defined AD (Braak stage III–VI) donors (total of 49 brain samples) (Supplementary Fig. 13, Supplementary data S2). Cortical samples from three regions were prepared from each brain to characterise pathology and transcript expression.

### Immunohistochemistry (IHC)

IHC (Table 1) was performed on FFPE sections (*n* = 49) from the EC, MTG and SSC of each brain studied and paired with material from the cryopreserved contralateral hemisphere used for nuclear preparations for snRNA sequencing. Standard immunostaining procedures as recommended by the manufacturer were followed using the ImmPRESS Polymer (Vector Laboratories) and Super Sensitive Polymer-HRP (Biogenex) kits. Briefly, after dewaxing and rehydration of slides, endogenous peroxidase activity was blocked with 0.3% H_2_O_2_, followed by antigen retrieval. For immunostaining using ImmPRESS kits, non-specific binding was blocked using 10% normal horse serum. Primary antibodies were incubated overnight at 4 °C. Species-specific ImmPRESS or Super Sensitive kits and DAB were used for antibody visualisation. Tissue was counter-stained by incubation in Mayer’s haematoxylin (TCS Biosciences) for 2 min, followed by dehydration, clearing and mounting.

**Table 1:** Antibodies used for immunohistochemistry

Antibody	Supplier	Dilution	Antigen retrieval	Staining kit
β-Amyloid (4G8)	Biolegend, 800,711	1:15,000	Steamer, CB pH6	Super Sensitive Polymer-HRP

*CB* citrate buffer, *EDTA* ethylenediaminetetraacetic acid

### Image analysis

Digital images were generated from IHC stained slides scanned using a Leica Aperio AT2 Brightfield Scanner (Leica Biosystems). Images were analysed using Halo software (Indica Labs) after optimisation of Indica Labs macros. Data from all cortical regions in each brain were combined.

### Imaging mass cytometry (IMC)

FFPE 5–10 μm sections were immunostained using lanthanide tagged antibodies before ablation. The slides underwent routine dewaxing and rehydration before undergoing antigen retrieval, in a pH8 ethylenediaminetetraacetic acid (EDTA) buffer. The slides were then treated with a 10% normal horse serum (Vector Laboratories) blocking solution before incubation with an antibody cocktail at 4 °C overnight. The slides were washed in 0.02% Triton X-100 (Sigma–Aldrich) before incubation with the Iridium-intercalator (Fluidigm) then washed and air-dried. All antibody conjugation was performed using the Maxpar X8 protocol (Fluidigm).

IMC was performed using a Hyperion Tissue Imager (Standard BioTools, San Francisco, USA) coupled to a Helios mass cytometer. The instrument was first tuned using the manufacturer’s 3-Element Full Coverage Tuning Slide before the slides were loaded into the device. Four 500 × 500μm^2^ regions of interest (ROI) within the grey matter were selected and ablated using a laser operating at a frequency of 200 Hz with 1 μm resolution. The data was stored as.mcd files compatible with MCD Viewer software (Fluidigm) and exported as TIFF files.

### Co-localisation of IMC markers for cohort-1

For the first IMC experiment (Cohort-1, Fig. 1a, b), the panel included antibodies listed in Table 2. The IMC channels which define a cell type were selected for masking, namely Iba1, GFAP, OLIG2, GLUT1, and MAP2. ImageJ (1.53c) was used for threshold correction and the despeckle function to reduce background noise. All of these channels were merged into a single field, alongside a DNA channel. The DNA channel was rendered red, while the cell type channels were rendered green before being saved as a JPEG. This JPEG was then used in Ilastik (1.1.3post3) using the pixel classification tool, this was then used to create a probability map clearly defining the images nuclei, cell signal and background. Finally, this probability map was imported to CellProfiler (4.2.1) for masking. Once masked the sample was opened in HistoCAT (1.76) and the masked cell data was exported as a csv, with quantitative values for the signal of each IMC channel for each cell.

**Table 2:** Antibodies used for imaging mass cytometry for cohort-1

Antibody	Gene name	Manufacturer	Catalouge	Metal-conjugate
β-Amyloid (4G8)		Biolegend	800,702	144 Nd
Iba1	AIF1	WAKO	019–19741	169 Tm
GFAP	GFAP	Abcam	ab218309	162 Dy
MAP2	MAP2	Abcam	ab236033	160 Gd
OLIG2	OLIG2	Abcam	ab220796	156 Gd
GLUT1	SLC2A1	Abcam	ab252403	176 Yb
p16	CDKN2A	Abcam	ab54210	141 Pr
GLB1	GLB1	Thermofisher	PA5-64,417	161 Dy
DNA				191 Ir

For peri-plaque microglia calculation (Fig. 4a, b, Supplementary data S1), the IMC channels Iba1, 4G8, p16 and GLB1 were saved as individual JPEGs for each sample using the ImageJ (1.53c) software, these images were then imported to Ilastik (1.1.3post3) were the cell signal and background were clearly identified as probability maps. These probability maps were then imported into CellProfiler (4.2.1). Each image was masked to identify cell signal as primary objects. The 4G8 primary objects were dilated by 10 µm to encompass any high proximity microglia. The Iba1, p16 and GLB1 objects were then merged, keeping only overlapping objects to identify either p16^+^, GLB1^+^ or p16^+^GLB1^+^ positive microglia, the masked cell data was exported as a csv, with quantitative values for the signal of each IMC channel for each cell.

### Automated IMC image analysis for cohort-2

For the second IMC experiment (Cohort-2, Fig. 1c–e, Supplementary data S2), the panel included antibodies listed in Table 3. The SIMPLI [7] pipeline (v. 1.1.0) was used for automated image processing and analysis for cohort-2 (4 ROI per section taken from 49 different brain blocks resulting in 194 QC-passed ROIs). This includes image processing (extraction of single.txt files from each ROI to TIFF images), normalisation and pre-processing using CellProfiler, where threshold smoothing scale, correction factor, lower and upper bounds, and manual threshold in were adjusted for each channel to remove background and keep specific signal only. Single-nuclei segmentation was performed within SIMPLI based on the intercalator (191Ir/193Ir) channel using StarDist, with the “2D_versatile_fluo” model and a probability threshold of 0.05. Single-nuclei channels intensity was used by SIMPLI for masking all detected nuclei to identify those expressing at least one of the cell type markers used (Iba1, OLIG2, MAP2, GFAP, GLUT1). Seurat, included in SIMPLI, was then used for unsupervised clustering of the identified cells (resolution 0.8). Resulting clusters were assigned to cell types based on cell type markers expression: 4 GLUT1 + clusters were assigned to endothelial cells (cluster 0, 11, 13, 14), 2 Iba1 + clusters to microglia (cluster 1, 9), 4 OLIG2 + clusters to oligodendrocytes (cluster 2, 3, 6, 12), 1 GFAP + cluster to astrocytes (cluster 7) and 4 MAP2 + clusters to neurons (cluster 4, 5, 8, 10). Cluster that expressed more than one marker were excluded from downstream analysis (cluster 10, 12, 14) with the exception of cluster 7 which was the only cluster expressing GFAP.

**Table 3:** Antibodies used for imaging mass cytometry for cohort-2

Antibody	Gene name	Manufacturer	Catalogue	Metal-conjugate
β-Amyloid (4G8)		Biolegend	800,702	144 Nd
Iba1	AIF1	WAKO	019–19741	169 Tm
GFAP	GFAP	Abcam	ab218309	162 Dy
MAP2	MAP2	Abcam	ab236033	160 Gd
OLIG2	OLIG2	Abcam	ab220796	156 Gd
GLUT1	SLC2A1	Abcam	ab252403	176 Yb
p21	CDKN1A	Abcam	ab218311	158 Gd
p16	CDKN2A	Abcam	ab54210	141 Pr
GLB1	GLB1	Thermofisher	PA5-64,417	161 Dy
yH2Ax	H2AX	Novus	NB100-384	152 Sm
Ki67	MKI67	Fluidigm	3168022D	168 Er
DNA				191 Ir

### Calculation of senescent nuclei in IMC for cohort-2

First, mean senescence marker (GLB1, p16, p21, γ-H2AX) expression was calculated per ROI and ROIs with the lowest 1% expression were excluded. Cells with the lowest 1% cell marker (Iba1, GFAP, MAP2, OLIG2, GLUT1) expression were removed from each ROI. The number of cells were then summed up across the ROIs at the sample level. The final numbers of ROIs per sample ranged between 3 and 4. Samples with < 3 cells per cluster were removed from subsequent analysis. Proportion of senescent nuclei per sample was then calculated by taking the ratio of cells co-expressing cell and senescence markers to cells positive for cell markers only. A Wilcoxon rank-sum test was performed to compare the differences of senescent nuclei proportion between AD and NDC across all clusters stratified by brain region and the effect sizes was calculated by the rank-biserial correlation [37]. The Wilcoxon rank-sum test was performed by wilcox.test() function, the *p* value and the 95% confidence intervals were calculated by rank_biserial() function from effect size (v. 0.8.6) package in R.

### Single nucleus isolation

Single nucleus isolation was conducted on 49 tissue blocks from (*n* = 24, NDC; *n *= 25, AD). Two samples were not available for sample preparation. Homologous fresh frozen brain tissue blocks from the EC, MTG and SSC were cryosectioned at 80 um and 200 mg of grey matter was collected in RNAse-free Eppendorf tube, as previously described [66]. Nuclei were isolated as previously described [66] using a protocol based on [40]*.* All steps were carried out on ice or at 4 °C. Tissue was homogenised in buffer (1% Triton X-100, 0.4 U/μl RNAseIn + 0.2 U/μl SUPERaseIn, 1 μl 1 mg/ml DAPI) using a 2 ml glass douncer. The homogenate was centrifuged at 4 °C for 8 min, 500 g and supernatant removed. The pellet then was resuspended in homogenisation buffer and filtered through a 70 um filter followed by density gradient centrifugation at 13,000 × *g* for 40 min. The supernatant was removed and nuclei were washed and filtered in PBS buffer (PBS + 0.5 mg/ml BSA + 0.4 U/μl RNAseIn + 0.2 U/μl SUPERaseIn). Nuclei were pelleted, washed twice in PBS buffer and resuspended in 1 ml PBS buffer. 100 μl of nuclei solution was set aside on ice for single nuclear processing.

### Single nucleus processing and sequencing

Isolated nuclei stained with Acridine Orange dye were counted on a LUNA-FL Dual Fluorescence Cell Counter (Logos Biosystems, L20001). Approximately 7000 nuclei were used for 10 × Genomics Chromium Single Cell 3′ processing and library generation. All steps were conducted according to the 10 × Genomics Chromium Single Cell 3′ Reagent Kits v3 User Guide, with 8 cycles of cDNA amplification until fragmentation, where 25 ng of amplified cDNA per sample was taken through for fragmentation. The final index PCR was conducted at 14 cycles. cDNA and library prep concentration were measured using Qubit dsDNA HS Assay Kit (ThermoFisher, Q32851) and DNA and library preparations were assessed using the Bioanalyzer High-Sensitivity DNA Kit (Agilent, 5067–4627). Pooled samples at equimolar concentrations were sequenced on an Illumina HiSeq 4000 according to the standard 10X Genomics protocol.

### Pre-processing and quality-control of snRNA sequencing data

Alignment and demultiplexing of raw sequencing data was performed using 10X Genomics Cell Ranger v3.1, with a pre-mRNA GRCh38 genome reference including both introns and exons. Downstream primary analyses of gene-cell matrices were performed using our scFlow pipeline [38]. Ambient RNA profiling was performed using emptyDrops with a lower parameter of < 100 counts, an alpha cutoff of ≤ 0.001, and with 10,000 Monte-Carlo iterations [45]. Cells containing ≥ 200 total counts or total expressive features, where expressivity was defined as a minimum of 2 counts in at least 3 cells, were included. An adaptive threshold was used to exclude nuclei with more than 4 median absolute deviation (MAD) total counts or total expressive features within a sample. The maximum proportion of counts mapping to mitochondrial genes was set to 5%. Doublets were identified using the DoubletFinder algorithm, with a doublets-per-thousand-cells increment of 8 cells (recommended by 10X Genomics), a pK value of 0.005, and embeddings were generated using the first ten principal components calculated from the top 2000 most highly variable genes (HVGs) [50].

### Integration, clustering, and visualization of data

The linked inference of genomic experimental relationships (LIGER) package was used to calculate integrative factors across samples [77]. LIGER parameters used included: k: 20, lambda: 5.0, thresh: 0.0001, max_iters: 100, knn_k: 20, min_cells: 2, quantiles: 50, nstart: 10, resolution: 1, num_genes: 3000, center: false. Two-dimensional embeddings of the LIGER integrated factors were calculated using the uniform-manifold approximation and projection (UMAP) algorithm with the following parameters: pca_dims: 30, n_neighbours: 70, init: spectral, metric: euclidean, n_epochs: 200, learning_rate: 1, min_dist: 0.7, spread: 0.85, set_op_mix_ratio: 1, local connectivity: 1, repulsion_strength: 1, negative_sample_rate: 5, fast_sgd: false [50]. The Leiden community detection algorithm was used to detect clusters of cells from the 2D UMAP (LIGER) embeddings; a resolution parameter of 0.0001 and a *k* value of 40 was used [70].

### Assigning cell type labels to snRNAseq cells

Automated cell-typing was performed essentially as previously described using the Expression Weighted Celltype Enrichment (EWCE) algorithm in scFlow against a previously generated cell-type data reference from the Allan Human Brain Atlas [38, 65]. The top five marker genes for each automatically annotated cell-type were determined using Monocle 3 and validated against canonical cell-type markers [71].

### Differential gene expression analysis

We used model-based analysis of single-cell transcriptomics (MAST) to identify genes differentially expressed (associated) between AD and NDC and with histopathological features (using 4G8 amyloid), using each feature as a dependent variable in a zero-inflated regression analysis using a mixed-model [20]. Data from both regions was combined. Additionally, diagnosis (control, AD) was used as a dependent variable to identify DGE between experimental groups. Models were fit separately for each cell-type. The model specification was zlm(~ dependent_variable + (1|sample) + cngeneson + pc_mito + sex + brain_region + age + PMI, method = “glmer”, ebayes = F). The fixed-effect term cngeneson is the cellular detection rate as previously described, and pc_mito accounts for the relative proportion of counts mapping to mitochondrial genes. Each model was fit with and without the dependent variable and compared using a likelihood ratio test. Genes expressed in at least 10% of cells (minimum of 2 counts per cell) were evaluated for gene expression. The threshold for significant differential gene expression was a log2 fold-change of at least 0.25 and an adjusted *p* value < 0.05.

### Impacted pathway analysis

Impacted pathway analysis (IPA) was performed essentially as previously described using the enrichR packages in scFlow [12, 44]. Statistically significant differentially expressed genes were submitted for IPA with the over-representation analysis (ORA) enrichment method against the ‘GO_Biological_Process’ and ‘KEGG’ databases. The false-discovery rate (FDR) was calculated using the Benjamini–Hochberg method and filtering was applied at a significance threshold of  ≤ 0.05.

### Gene set score visualisation

Dot plot (Fig. 2c) was generated using Seurat DotPlot() function using CSP, SIP and custom list (Supplementary data table). Genes expressed in less than 10% of any cell population were excluded. Gene set or module feature plots were generated by first calculating aggregated gene set scores using AddModuleScore() function from Seurat and then plotted using FeaturePlot scCustom() from scCustomize (v. 2.0.1).

### Gene set enrichment analyses (GSEA)

Gene sets were collected either from published literature or from publicly available databases. “canonical senescence pathway”, “senescence initiating pathway” and “senescence response pathway” was collected from [16]. A custom set of senescence genes were used in Fig. 2c and S3a. Additional pathways were selected from Gene Ontology (GO) and KEGG databases using the search terms “senescence”, “endoplasmic reticulum stress”, “endosome”, “endosomal”, “lysosome”, “lysosomal”, “mitochondrial” and “oxidative stress”. Pathways with less than 10 genes were excluded. Pathways that included positive or negative regulation in the description also were excluded from the resultant term list, yielding 33 biological processes (Supplementary data S5). We then used AUCell (R package v1.6.1) to quantify the expression of the gene set signature in each nucleus. Normalised data were processed in AUCell using the AUCell_build. Rankings function to generate ranking of each gene. The resulting rankings, along with the gene lists of interest, were then run by the function AUCell_calcAUC (aucMaxRank set to 5% of the number of input genes) to generate AUC scores of the gene in each nucleus. We then used the dream function from variancePartition package in R [31] to compare the expression changes across the categorical variables or regressed against β-amyloid densities. We used a linear mixed-model where individual sample “manifest” were set as the random effect to avoid the pseudoreplication bias and the following covariates (total_features_by_counts, pc_mito, sex, brain_region, age and PMI) were set as fixed effects.

### Quantification of senescent nuclei fraction in snRNAseq data using gene set score

Gene set scores for the “canonical senescence pathway (CSP)” was calculated per nucleus as described in the previous section. Cells were considered senescent if the gene set score was > median + 3 MADs (median absolute deviation).

### Meta-analysis

Publicly available snRNA sequencing data (Supplementary data S8) were downloaded with appropriate authorization where required. Individual datasets were processed uniformly with similar parameters using nf-core/scflow as described above. For meta-analysis, microglial clusters were isolated, “canonical senescence pathway” gene set scores were calculated per nuclei as described above. Gene set scores were then averaged across all the nuclei per sample and meta-analysis was performed using the runMetaAnalysis() function from R package MetaIntegrator (v. 2.1.3). Meta-analysis result was visualised as a forest plot generated from R package forestplot (v. 3.1.1).

### Sub-clustering and annotation

We generated transcriptomically defined subsets for each of the glial cell type clusters (astrocytes, microglia and oligodendrocytes). To do this, individual cell type clusters were first normalized and scaled using Seurat’s NormalizeData and ScaleData functions, respectively. RunPCA function was used to calculate the first 20 PCs using the top 2000 highly variable genes. Individual samples were re-integrated using Harmony [39], using Seurat’s RunHarmony() function (group.by.vars = "manifest"). To produce the final UMAP, we used the following parameters in RunUMAP() (dims = 1:20, n.epochs = 200). To identify clusters, we used first the function FindNeighbors() (dims = 1:20) and then performed unbiased clustering using FindClusters() (resolution = 0.5). To annotate the sub-population of microglia, we used the gene sets from [47].

### Pseudo-time

Pseudo-time analysis was performed to infer the phenotypic transitions happening between the different sub-population of each glial cell types. Unsupervised single-cell trajectory analysis was performed with *Monocle3*, an algorithm that allows to learn the sequence of gene expression changes each cell must go through as part of a dynamic biological process. We used *SeuratWrappers* to convert our *Seurat* object into a *Monocle* object with *as*.*cell_data_set()*. We kept the UMAP embeddings previously calculated with *RunUMAP()* in order to estimate the phenotypic transitions between our annotated cell states. We run *cluster_cells()* and *learn_graph()* (resolution = 0.001, use_partition = F, *close_loop* = *F, learn_graph_control* = *list(rann*.*k* = *100, prune_graph* = *TRUE, orthogonal_proj_tip* = *F, minimal_branch_len* = *10, ncenter* = *300)*) to learn the trajectory.

### Supplementary Information

Below is the link to the electronic supplementary material.

Supplementary file1 (DOCX 20 KB)

Supplementary file2 (TIFF 96263 KB)

Supplementary file3 (TIFF 99463 KB)

Supplementary file4 (TIFF 89097 KB)

Supplementary file5 (TIFF 95379 KB)

Supplementary file6 (TIFF 105072 KB)

Supplementary file7 (TIFF 79219 KB)

Supplementary file8 (TIFF 73157 KB)

Supplementary file9 (TIFF 92278 KB)

Supplementary file10 (TIFF 70662 KB)

Supplementary file11 (TIFF 87960 KB)

Supplementary file12 (TIFF 29874 KB)

Supplementary file13 (TIFF 92583 KB)

Supplementary file14 (TIFF 72 KB)

Supplementary file15 (xlsx 12613 KB)

## Data Availability

The processed snRNAseq data is available to researchers for download from the Gene Expression Omnibus (GEO) database (https://www.ncbi.nlm.nih.gov/geo/) under the id GSE264648. The raw snRNAseq data can be accessed from https://www.synapse.org/#!Synapse:syn36812517/wiki/619350.

## Code availability

All codes are available upon direct contact to the lead author.
