# Supplementary material for: Characterisation of premature cell senescence in Alzheimer’s disease using single nuclear transcriptomics
Source: Acta Neuropathol. 2024 May 2;147(1):78. doi: 10.1007/s00401-024-02727-9 (PMC11065703; doi:10.1007/s00401-024-02727-9)
Supplement: Supplementary file 1 — Supplementary file1 (DOCX 20 KB) [file 401_2024_2727_MOESM1_ESM.docx]

**Supplementary figure 1: Representative IMC images showing overlapping expression of cell type and senescence markers.** Multiplexed imaging mass cytometry (IMC) (scale bar = 50 µm) revealed overlapping expression of a) microglia (IBA1), b) oligodendrocyte lineage (OLIG2) and c) astrocyte (GFAP) markers with senescence markers (GLB1 and p16). Orange arrowheads highlight senescent cells. d) Proportions of GLB1^+^ endothelial cells (GLUT1^+^) and neurons (MAP2^+^) were calculated in AD and NDC using IMC.

**Supplementary figure 2: Cells expressing senescence markers in entorhinal and somatosensory cortices detected by IMC**. a) Multiplexed imaging mass cytometry (IMC) revealed overlapping expression of DNA damage marker γH2AX and cell type specific markers (IBA1; microglia, MAP2; neuron, GFAP; astrocyte). Proportion of nuclei of all clusters expressing senescence markers in AD and NDC in b) entorhinal cortex (EC) and c) somatosensory cortex (SSC) (Wilcoxon rank-sum test, p <= 0.1 is reported).

**Supplementary figure 3: All cell clusters are equally represented in both AD and NDC groups**. a) UMAP showing the cellular clusters generated by SIMPLI from cohort-2 IMC data grouped by Control or AD in each brain regions. b) Proportions of all cell clusters identified by SIMPLI are equally represented in AD and Control.

**Supplementary figure 4: Canonical marker gene expression reveals distinct cell types. a)** UMAP featureplots of canonical cell marker genes for astrocytes (GFAP), microglia (CSFR1), oligodendrocytes (PLP1), oligodendrocyte precursor cells (PDGFRA), vascular cells (CLDN5, COL4A1, DCN) and neurons (GAD1, GAD2, CUX2, RORB, SST, PVALB, SV2C, VIP). b) Percentage of senescent nuclei between AD and NDC across all cell types (Wilcoxon rank-sum test, Astro, astrocytes; Micro, microglia; Oligo, oligodendrocytes; OPC, Oligodendrocyte progenitor cells; Vasc, vascular cells; Exc, excitatory neurons, Inh; Inhibitory neurons).

**Supplementary figure 5: Differential gene and pathway expression between AD and NDC cortical brain tissue for nuclei from different cell types.** Volcano plot showing differentially expressed genes in AD compared to NDC at logFC cut-off 0.25 and adjusted p-value cut-off 0.05 for a) astrocytes, b) oligodendrocytes, c) OPC, d) excitatory neurons and e) inhibitory neuron and f) vascular cells. Orange and blue bar plots are show major gene ontologies (GO Biological Processes) for upregulated or downregulated genes, respectively.

**Supplementary figure 6: Gene set enrichment analysis associated with increasing 4G8+ β-amyloid load.** a) Heatmap showing z-scores for enrichments in senescence gene sets associated with the same measures of β-amyloid pathology load across cell types. b, c) CSP gene set score per sample is plotted as a function of b) %4G^+^ area (β-amyloid) densities and c) braak stages. Linear mixed model, adjusted pval is reported.

**Supplementary figure 7: Meta-analysis of senescence-associated gene expression in previously published AD snRNAseq datasets.** We re-analysed previously published datasets [66]) to explore senescence-associated gene expression upregulated in a) astrocytes or b) microglia with increasing %4G8^+^ (β-amyloid) load. c) Forest plot showing the meta log2FC (orange diamond), meta p-value and log2FC effect sizes (red square) along with 95% CI (error bars) of “canonical senescence pathway” gene set expression between AD and NDC samples in individual datasets calculated from the meta-analysis of 14 previously published AD snRNAseq datasets. d) Contrast of the mean CSP gene set scores as a function of age for AD and NDC samples. Linear plateau modelling, pval is reported.

***Supplementary figure 8: Gene Ontology (GO) pathway enrichment in genes up- and down-regulated with greater 4G8^+^ β-amyloid areas in tissue sections paired to those for snRNAseq.*** *Top significantly enriched pathways from regression of gene expression against %4G8^+^(β-amyloid) area for a) upregulated and b) downregulated genes from each cell type. Pathways were considered significant at 0.05 FDR.*

***Supplementary figure 9: Senescence-associated gene signatures were differentially expressed for different microglia sub-types.*** *a) Heatmap showing the top 5 marker genes distinguishing microglial sub-types. b) Mean expression of marker gene sets* [47] *distinguishing microglial sub-populations. c) Enrichment of marker gene sets from* [23] *in the markers detected in microglial sub-populations in this study. d, e) UMAP color-scaled featureplots showing total numbers of genes expressed (d) and the percentages of mitochondrial genes (e) in microglia nuclei highlighting that increased relative proportions of senescence genes are expressed in nuclei expressing lower mitochondrial gene numbers. f, g) Percentages of senescent nuclei in AD and NDC (f) and as a function of 4G8^+^ β-amyloid load (g) for the major microglial sub-types stratified by brain region. h, i) Boxplots showing the scaled mean expression of canonical senescence gene set across microglial sub-population grouped by h) CD33 variants and i) APOE variants.*

**Supplementary figure 10: Oligodendrocyte and astrocyte subcluster characterisation**. a) Heatmap showing the top 5 marker genes distinguishing oligodendrocytes subpopulations. b) Dotplot showing enrichment of external marker genes [33, 48] for oligodendrocyte sub-clusters across the sub-population identified in this study. c) Heatmap showing the top 5 marker genes distinguishing astrocyte subpopulations.

**Supplementary figure 11: Microglial sub-population changes as a function of age**. a, b) Percentages of nuclei for microglial sub-populations as a function of donor age at death in this study cohort-2 (a) and in the integrated data used for the meta-analysis (b).

**Supplementary figure 12: Microglia trajectory module featureplots**. a) Module featureplots showing the expression of gene modules derived from microglial trajectory analyses. b) Module-5 gene set expression as a function of %4G8^+^ densities.
