# Supplementary figures and images for: Characterisation of premature cell senescence in Alzheimer’s disease using single nuclear transcriptomics
Source: Acta Neuropathol. 2024 May 2;147(1):78. doi: 10.1007/s00401-024-02727-9 (PMC11065703; doi:10.1007/s00401-024-02727-9)

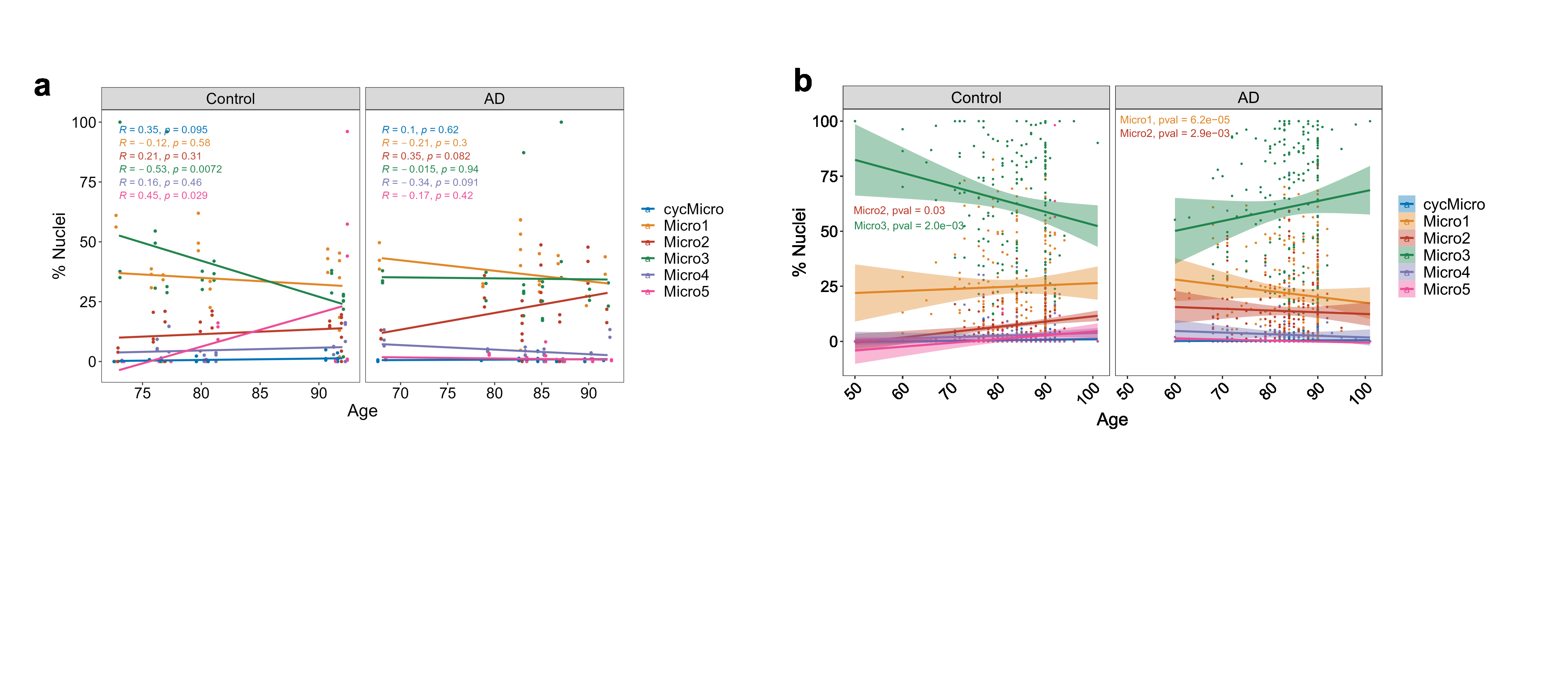

Supplement: Supplementary file 12 — Supplementary file12 (TIFF 29874 KB) [file 401_2024_2727_MOESM12_ESM.tiff]

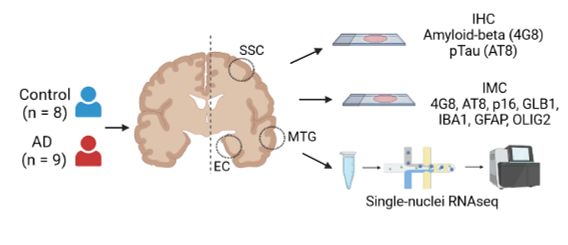

Supplement: Supplementary file 14 — Supplementary file14 (TIFF 72 KB) [file 401_2024_2727_MOESM14_ESM.tiff]
